# Injectable SN-38-embedded Polymeric Microparticles Promote Antitumor Efficacy against Malignant Glioma in an Animal Model

**DOI:** 10.3390/pharmaceutics12050479

**Published:** 2020-05-24

**Authors:** Yuan-Yun Tseng, Tao-Chieh Yang, Shu-Mei Chen, Shun-Tai Yang, Ya-Ling Tang, Shih-Jung Liu

**Affiliations:** 1 Division of Neurosurgery, Department of Surgery, Shuang Ho Hospital, Taipei Medical University, Taipei 11031, Taiwan; britsey@tmu.edu.tw (Y.-Y.T.); 08512@s.tmu.edu.tw (S.-T.Y.); 2 Department of Surgery, School of Medicine, College of Medicine, Taipei Medical University, Taipei 11031, Taiwan; 3 Department of Neurosurgery, Chung Shan Medical University Hospital, Taichung 40201, Taiwan; cshy1801@csh.org.tw; 4 Department of Neurosurgery, Taipei Medical University Hospital, Taipei Medical University, Taipei 11031, Taiwan; 191001@h.tmu.edu.tw; 5 Department of Mechanical Engineering, Chang Gung University, Taoyuan 33302, Taiwan; m0722005@cgu.edu.tw; 6 Department of Orthopedic Surgery, Chang Gung Memorial Hospital-Linkou, Taoyuan 33305, Taiwan

**Keywords:** malignant glioma (MG), 7-ethyl-10-hydroxycamptothecia (SN-38), irinotecan (CPT-11), poly(lactide-*co*-glycolide) (PLGA), intratumoral drug delivery

## Abstract

Malignant glioma (MG) is extremely aggressive and highly resistant to chemotherapeutic agents. Using electrospraying, the potent chemotherapeutic agent 7-ethyl-10-hydroxycamptothecia (SN-38) was embedded into 50:50 biodegradable poly[(d,l)-lactide-*co*-glycolide] (PLGA) microparticles (SMPs). The SMPs were stereotactically injected into the brain parenchyma of healthy rats and intratumorally injected into F98 glioma-bearing rats for estimating the pharmacodynamics and therapeutic efficacy. SN-38 was rapidly released after injection and its local (brain tissue) concentration remained much higher than that in the blood for more than 8 weeks. Glioma-bearing rats were divided into three groups—group A (*n* = 13; stereotactically injected pure PLGA microparticles), group B (*n* = 12; stereotactically injected Gliadel wafer and oral temozolomide), and group C (*n* = 13; stereotactic and intratumoral introduction of SMPs). The SMPs exhibited significant therapeutic efficacy, with prolonged survival, retarded tumor growth, and attenuated malignancy. The experimental results demonstrated that SMPs provide an effective and potential strategy for the treatment of MG.

## 1. Introduction

The most prevalent and lethal primary brain tumor, malignant glioma (MG), is currently incurable and patients with MG have an average life span of less than 12 months after diagnosis [1,2]. Utmost operative removal of the tumor followed by standard radiotherapy with adjuvant and concurrent chemotherapy is the prevalent therapeutic strategy for clinically treating MG, in patients younger than 70 years [3,4]. Despite intensive effort being expended over the past 30 years to discover efficient chemotherapeutic agents for MG, the efficacy of MG therapy has not improved appreciably [4,5].

Due to the frequent unsuccessful clinical trials for MG treatments, the role of chemotherapy has long been questioned. More than 98% of the analyzed agents cannot cross the blood–brain barrier (BBB), mainly because of their less-than-optimal physicochemical or molecular properties [6]. The BBB restrains the transport of chemotherapeutic agents inside brain lesions, even for agents at toxic systemic levels [2,7]. This high resistance to chemotherapeutic agents remains the major cause of treatment failure [5,8]. Although considerable research has been conducted on novel treatments for MG, alkylating agents remain the foundation of chemotherapy for MG. The US Food and Drug Administration have approved alkylating agents such as temozolomide (TMZ) and polifeprosan 20 with bis-chloroethylnitrosourea (BCNU) (administered through a Gliadel polymer implant wafer) for use in MG treatment. Nevertheless, the most critical limitation of alkylating agents for treating MG is that cancer cells develop de novo or acquired resistance [9,10]. More than 90% of patients with recurrent gliomas do not respond to the second cycle of TMZ chemotherapy [11]. Chemotherapeutics impair the cerebral parenchyma, leading to systemic toxicity and adverse influences; these effects notably limit the maximum tolerated dose and thus the drugs’ treatment competence [9,12].

Irinotecan hydrochloride (CPT-11) is a water-soluble derivative of camptothecin (CPT) and is administrated clinically to treat malignant lymphoma and colorectal, gastric, lung, ovarian, uterine cervical, and other cancers [13]. In animal studies, CPT-11 has exhibited antitumor activity against a broad range of subcutaneous and intracranial human gliomas, ependymomas, and medulloblastomas [1,14]. After intravenous administration, irinotecan is metabolized in the liver to produce the active metabolite 7-ethyl-10-hydroxycamptothecia (SN-38) [1,14]. CPT-11 and SN-38 act as topo-I inhibitors that stabilize the complex between topo-I and DNA, which conflicts with the moving DNA replication forks, eventually leading to impairment of the double-stranded DNA [15,16]. SN-38 is 1000-times more potent than CPT-11, and its half-life is longer than those of topotecan and CPT-11 [17,18]. Therefore, direct use of SN-38 might be more effective than topotecan and CPT-11, for treating MG. However, clinical utilization of SN-38 is restricted because SN-38 is highly toxic when administered intravenously [17,19], even after various efforts to modify the water insoluble drug/molecules with formulation using liposome or other polymer to increase cellular uptake, bioavailability, etc. [20,21].

In this study, we developed SN-38 loaded biocompatible and biodegradable poly(lactid-*co*-glycolid) (PLGA) microparticles (SMPs) through the electrospraying technique. The sizes of the SMPs were tiny and could be mixed with solution and introduced into brain parenchyma via stereotactic techniques, instead of surgical craniotomy. Local and direct injection of SMPs to the target site can provide the advantages of achieving sustained/controlled drug release, high anticancer activity, and low systemic side effects. Furthermore, stereotactic injection can be repeated safely and is considered the procedure of choice in cases of multiple or deep tumors, for which an open surgical approach is impossible or is perilous, especially when the tumor is located at crucial areas of the brain. After electrospraying, the in vitro release profiles of SN-38 from SMPs was characterized using an elution method. SMPs were then stereotactically introduced into the cerebral parenchyma of healthy rats and the tumors of tumor-bearing rats, by using a syringe following a simple burr hole surgery. The in vivo SN-38 release characteristics were determined and therapeutic efficacy was evaluated and compared with other treatment regimens.

## 2. Materials and Methods

### 2.1. Chemical and Reagents

Poly(lactid-*co*-glycolid) polymers with a lactide:glycolide ratio of 50:50 (Resomer^®^ RG503) were acquired from Boehringer Ingelheim (Ingelheim am Rhein, Germany), and SN-38 was purchased from Sigma Aldrich (Saint Louis, MO, USA). Oral TMZ (20 mg) was purchased from Rising Pharmaceuticals, Inc. (East Brunswick, NJ, USA). Pathological examinations, including hematoxylin and eosin (H&E) staining, glial fibrillary acidic protein expression (GFAP) and Ki-67 labeling index were carried out by a professional analysis service (Biotools Co., New Taipei, Taiwan).

### 2.2. Fabrication of the SMPs

Both virgin PLGA microparticles and SMPs were prepared using a laboratory-made electrospraying device, featuring a syringe and needle (internal diameter = 0.60 mm), ground electrode, collector, and high voltage supply. To prepare the virgin PLGA particles, 500 mg of PLGA were dissolved in 10 mL of dichloromethane for 1 h. During the electrospraying process, the solution was transported using a syringe pump with a volumetric flow rate of 1 mL/h. The needle was connected to a high voltage supply with a positive direct current voltage of 14 kV. The distance between the needle tip and ground electrode was 10 cm.

To manufacture the SMPs, 300 mg of PLGA and 50 mg of SN-38 were first mixed with 1 mL of dichloromethane for 1 h. The solution was then transported with a pump with a volumetric flow rate of 0.9 mL/h. The positive direct current voltage was 8 kV, while the distance between the needle tip and ground electrode was 13 cm. All electrospraying experiments were completed under a temperature of 25 °C and a relative humidity of 60%.

### 2.3. Fourier-Transform Infrared Spectroscopy

Fourier-transform infrared spectroscopy (FTIR) was employed to obtain spectra of pure PLGA microparticles and SMPs. The FTIR analysis was conducted using a Nicolet iS5 spectrometer (Thermo Fisher Scientific, Waltham, MA, USA) at a resolution of 4 cm^−1^ (32 scans). Electrosprayed particles were compressed into potassium bromide discs, and the spectra were recorded over the range 400–4000 cm^−1^.

### 2.4. Thermal Analysis

Thermal analysis was carried out using a differential scanning calorimetry assay (DSC) instrument Model DSC25 (TA Instruments, Delaware, USA). The DSC test runs were completed to characterize the thermal properties of SN-38, PLGA, and SMPs, with a heating rate of 10 °C/min and a temperature range of 25–250 °C.

### 2.5. Characterization of SMPs

The morphology of the electrosprayed microparticles was observed using field-emission scanning electron microscopy (SEM) performed with a JEOL Model JSM-7500F (JEOL, Tokyo, Japan). The size distribution of the electrosprayed particles was measured by an ELSZ-2000 particle size analyzer (Otsuka Electronics, Osaka, Japan).

### 2.6. In Vitro Elution Characteristics of SN-38

The levels of SN-38 eluted from the SMPs were characterized by an in vitro elution method. Fifteen milligrams of SMPs were placed in a test tube (N = 3) consisting of 1 mL of phosphate-buffered saline (PBS). The volume of PBS used here was to simulate that of the approximate brain fluid in the rats. The follow-up was placed in an incubator shaker (Deng-Yng Incubator E600, Taipei, Taiwan) with a speed of 100 rpm at 37 °C for 24 h, the eluent was collected and replaced by 1 mL of fresh PBS, for the following 24 h period. The procedure was carried out daily for eight weeks. The concentration of SN-38 in the eluent was examined utilizing a Hitachi L-2200 HPLC analysis (Multisolvent Delivery System, Tokyo, Japan). A Symmetry C8 (5 μm, 4.6 × 250 mm, Waters) column was used to separate the SN-38. The mobile phase contained NaH_2_PO_4_ (25 mM, pH = 3.1): acetonitrile (Sigma-Aldrich, Saint Louis, MO, USA) (50/50, *v/v*). The absorbency was monitored at a wavelength of 265 nm and a flow rate of 1.0 mL/min. The retention time was 3.62 mins. All experiments were performed in triplicates and the samples were diluted to bring the unknown concentrations into the range of the assay standard curve. A calibration curve was obtained for each set of measurements (correlation coefficient > 0.99). The eluted concentration was characterized employing the highly sensitive HPLC system.

### 2.7. Surgical Procedure

All procedures related to animals were approved by the Institutional Animal Care and Use Committee of Taipei Medical University (LAC-2018-0412, approved date: 4 March 2019), and the animals were kept in accordance with the guidelines of the Department of Health and Welfare, Taiwan. Effort was made to minimize the number of animals used and their suffering. A total of 90 male Sprague Dawley rats weighing between 200 and 250 g were commercially acquired from BioLASCO Taiwan Co., Ltd. (Taipei, Taiwan).

Forty rats were enrolled in the in vivo drug release study. All rats were anesthetized through inhalation of halothane. The corneal reflex and tail pinch tests were performed to monitor the depth of anesthesia. After the postorbital areas were shaved and sterilized, a small longitudinal scalp incision of approximately 8-mm length was performed lateral to the midline. The muscle and scalp fascia were dissected, and a burr hole (approximately 1.5 mm in diameter) was drilled using an electric burr. After meticulous hemostasis, the experimental rats were secured on a stereotactic instrument (Model: 68001, RWD Life Science Inc., San Diego, CA, USA). A 20-µL Neuros syringe was inserted to a depth of 3 mm below the brain surface in the center of the burr hole. Subsequently, 2.4 mg of biodegradable PLGA microparticles incorporated with 0.4 mg SN-38 (SMPs) were mixed with 10 µL of DMSO, and the mixture was injected slowly into the cerebral parenchyma (in total, the injection lasted >3 min), by using a syringe infusion pump. The operative wound was then sutured using 3-0 nylon. The rats that exhibited intraoperative brain injury or infection and died within 24 h after operations were excluded from the study.

### 2.8. In Vivo SN-38 Pharmacokinetics

**Figure 1:**
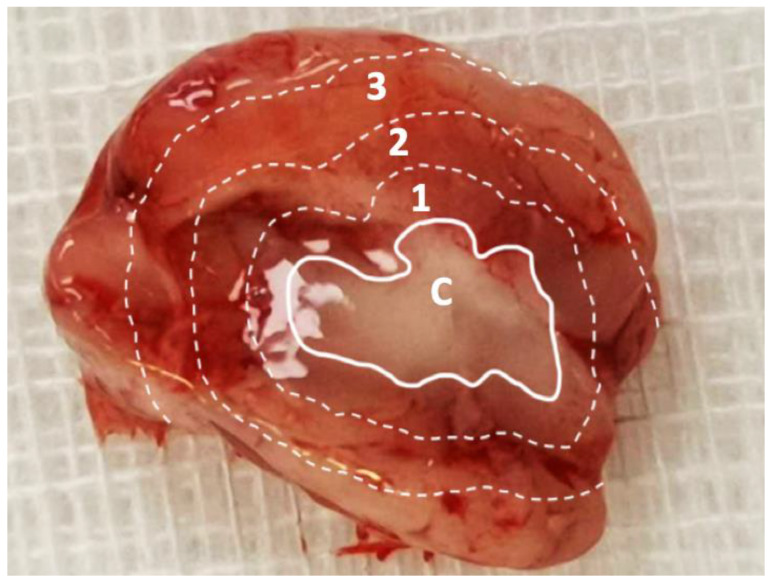
Division of sampling zone. The injected SN-38-embedded poly[(d,l)-lactide-*co*-glycolide] microparticles (SMPs) resulted in a white core, and the brain parenchyma was divided into zones 1–3 (each zone of thickness, ~2–3 mm) for tissue sampling. C: core, 1: zone 1, 2: zone 2, and 3: zone 3.

The rats were randomly divided into nine groups of three or four rats, with each group being euthanized after a certain period (3 days and 1, 2, 3, 4, 5, 6, 7, and 8 weeks). Carbon dioxide inhalation was used to euthanize the rats. Using 1-mL syringes through cardiac puncture, blood specimens (approximately 0.5 mL) were collected. Ipsilateral sections injected with SMPs was surgically extracted. Cerebral parenchymal specimens with dimensions of 8 × 8 mm^2^ and thicknesses of 8–10 mm were divided into three zones from the core (the tract of injected SMPs), outward from the center of the brain; the zones had a thickness of 2–3 mm (Figure 1). Approximately 50 mg of the cerebral parenchymal specimen was obtained from each zone. All samples (blood and various brain tissues) were collected on day 3 or weeks 1, 2, 3, 4, 5, 6, 7, or 8, depending on the group. The tissue specimens were extracted through sonication for 20 s and then centrifuged. Plasma was collected and maintained at −80 °C. The level of SN-38 in the collected solutions was evaluated using a Hitachi L-2200 HPLC assay (Multisolvent Delivery System, Tokyo, Japan).

### 2.9. Glioma Model Creation and Treatment

The rat glioma cell line F98 (ATCC-CRL-2948) was commercially acquired from the American Type Culture Collection. F98 glioma cells were cultured in the Dulbecco’s Modified Essential Medium (Sigma Aldrich, Saint Louis, MO, USA), augmented with 10% fetal bovine serum.

Adhering to the aforementioned procedure, the brains of another 50 healthy male Sprague Dawley rats were injected with 10 µL of isotonic saline containing 2–3 × 10^5^ F98 rat glioma cells. Approximately 12–14 days after injection of the tumor cell solution, the rats received a brain MRI scan (T1- and T2-weighted images, 7-Tesla Biospec, Bruker, Ettlingen, Germany) to verify that brain gliomas had developed (excluding infection and intracranial hemorrhage). The MG-bearing rats were anesthetized through halothane inhalation, and the scalp wound was reopened and positioned in a stereotactic instrument. The MG-bearing rats were then randomly divided into three groups. A Neuros syringe was inserted to a depth of 3 mm in the center of the previous burr hole, and 10 µL of DMSO solution mixed with pure 2.4 mg PLGA microparticles was injected into the mass of glioma in group A. In group B, a one-twentieth Gliadel wafer (containing 0.385 mg of BCNU) mixed with 10 µL of DMSO was intratumorally injected into the MG-bearing rats. TMZ (20 mg) was then orally administered at a dosage of 200 mg/body surface area (m^2^), once daily in the first 5 days; subsequently, TMZ administration was halted for 23 days (cycle 1). If the MG-bearing rats survived longer than 1 month, cycle 2 was begun. The MG-bearing rats in group C only received the injection of SMP/DMSO solution (2.4 mg of PLGA microparticles containing 0.38–0.4 mg of SN-38). General appearance, neurological deficit or seizure attack, diarrhea, and other abnormal finding were examined during the whole study period (80 days). T2-weighted MRI images were acquired to estimate the MG volumes at 1, 2, 4, 6, and 8 weeks posttreatment. The MG volumes were reconstructed and estimated using FDA-approved, open-source Digital Imaging and Communication in Medicine imaging OsiriX software. At least one rat in each group was sacrificed at 6 weeks posttreatment, and the brain parenchyma was meticulously extracted for pathological examination. The extracted cerebral parenchyma was fixed with 10% formalin and enclosed in paraffin. Subsequently, the brain tissue specimens were soaked in 10% buffered formal saline, followed by mounting in paraffin. They were then cut into sections of 5-µm-thick, H&E-stained, and evaluated microscopically. The Ki-67 labeling index, estimated by MIB-1 immunostaining, was denoted as a percentage, obtained by computing the amount of positively stained nuclei in 1000 tumor cells collected from 3–5 fields (each has an area of 0.16 mm^2^) that were observed at high-power amplification. Immunohistochemical staining of antibodies toward GFAP was also completed on these sections.

### 2.10. Statistical Analysis

Experimental data are displayed as mean ± standard deviation. By using commercially available Stata software (Version 12.0; Stata, College Station, TX, USA), statistically significant differences were determined using the paired-sample *t*-test. Statistical significance was defined as *p* < 0.05. The Kaplan–Meier method was employed to analyze the survival data, with statistical significance determined using the post-hoc log-rank test. A repeated-measures mixed model was used to evaluate the therapeutic effects of different treatments on tumor growth.

## 3. Result

### 3.1. Morphology of the Microparticles

**Figure 2:**
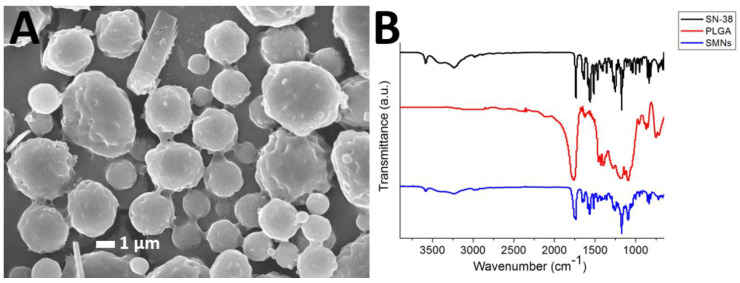
SN-38-embedded poly[(d,l)-lactide-*co*-glycolide] microparticles (SMPs) morphology. (**A**) Scanning electron microscopy (SEM) image of SMPs. The particles possess an average SMPs diameter of 1.58 ± 0.54 µm. (**B**) Fourier-transform spectra of the pharmaceuticals, pure poly[(d,l)-lactic-*co*-glycolide] microparticles, and SMPs.

Figure 2A presents an SEM image of the SMPs. The measured particle size and zeta potential and polydispersity index were 1.58 ± 0.54 µm, −0.86 ± 0.1 mV, respectively. The polydispersity index was 3.577. In addition, the cumulant diameter D10, D50, and D90 were 921 nm, 1.41 µm, and 2.23 µm, respectively.

### 3.2. FTIR Spectroscopy

Figure 2B displays the FTIR spectra of SN-38, pure PLGA microparticles, and SMPs. The vibration peak at 1660 cm^−1^ was attributed to the C=N bonds in SN-38. The new peak at 3010–3100 cm^−1^ of the SMPs spectrum could be attributed to the aromatic ring of the pharmaceuticals [22,23]. Furthermore, the absorption at 3350–3500 cm^−1^ was owing to the N-H bond of SN-38. Thus, the FTIR spectra revealed that SN-38 was successfully incorporated into the SMPs.

### 3.3. DSC Analysis

**Figure 3:**
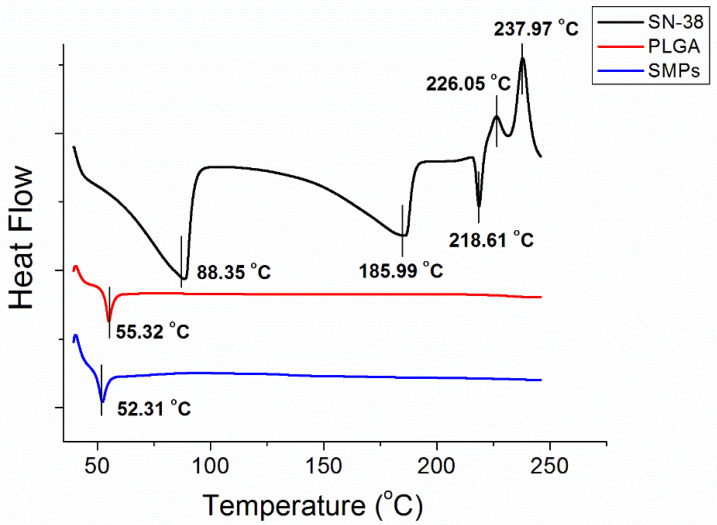
Thermal characteristic of SN-38, PLGA, and SN-38-loaded PLGA microparticles.

Figure 3 displays the thermal characteristics of SN-38, PLGA, and SN-38 loaded PLGA microparticles from the DSC analysis. SN-38 showed exothermic peaks at 88.4 °C, 186.0 °C, and 218.6 °C, and endothermic peaks at 226.0 °C and 238.9 °C, respectively. Pure PLGA exhibited an endothermic peak at 55.3 °C, corresponding to the glass transition temperature of the polymeric material. After the addition of SN-38, the SMPs displayed a slightly reduced peak at 52.3 °C, while all peaks from SN-38 vanished.

### 3.4. In Vitro Release Profiles of SN-38

**Figure 4:**
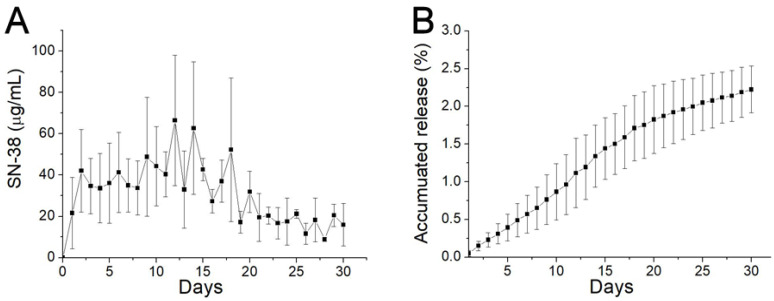
In vitro release. (**A**) Daily release; and (**B**) accumulative release of 7-ethyl-10-hydroxycamptothecia (SN-38) from biodegradable SMPs.

The in vitro daily and cumulative release curves of SN-38 from the SMPs are presented in Figure 4. The release profiles show a mild release at day 1 (with concentrations of 21.6 ± 17.4 µg/mL), followed by a sustained release stage in days 2–15 (with concentrations of 48.7 ± 28.7 µg/mL). The concentration then gradually diminished thereafter. Overall, due to the low water solubility of SN-38 (0.29 mg/mL, Sigma Aldrich), the release drugs remained low during the entire elution process.

### 3.5. In Vivo Characteristics of SN-38 Release from SMPs

**Figure 5:**
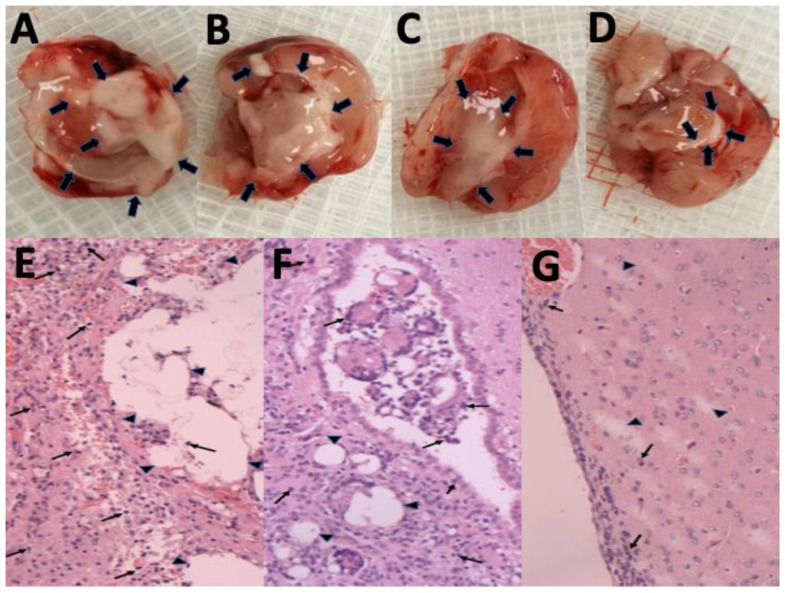
Degradation of SN-38-embedded poly[(d,l)-lactide-*co*-glycolide] microparticles (SMPs) and histological examination of brain tissue. Overall appearance at (**A**) 1 week, (**B**) 4 weeks, (**C**) 6 weeks, and (**D**) 8 weeks, after stereotactic SMP injection into the brain parenchyma. The injected SMPs (indicated with black arrows) were denser and greater in number during the first few weeks. Only a few SMPs were observed at the end of the study. Hematoxylin-and-eosin (H&E)-stained ipsilateral brain tissues obtained at (**E**) 1 week, (**F**) 4 weeks, and (**G**) 8 weeks. The SMPs (indicated by triangles) became larger and more concentrated, and gradually degraded over time. Only a few tiny residual particles were observed at 8 weeks postinjection. A focal and temporary inflammation reaction (accumulation of numerous inflamed leukocytes, indicated by small arrows) occurred at the injected area. No clear inflammation was observed in the noninjected areas in the histological examination. Magnification = 100×.

The gross appearance of cerebral parenchyma at 1, 4, 6, and 8 weeks after SMP injection is displayed in Figure 5A–D, respectively. The injected SMPs were denser and greater in the area in the first few weeks and degraded gradually. Only a few residual SMPs remained at the end of the study (8 weeks). The surrounding scalp and brain tissues were relatively clear, and no signs of inflammation or infection (discharge of pus or exudate, or granulation) were observed. Figure 5E–G display the H&E-stained sections of ipsilateral brain tissue obtained in the early (1 week), middle (4 weeks), and late (8 weeks) stages. The injected SMPs elicited focal inflammation in the SMP-injected regions because of the increased number of leukocytes, especially granulocytes, from the blood entering the injured tissues. No inflammation reaction appeared to occur in the noninjected areas. At week 4, the inflammation was reduced, and fewer granulocytes encircled the injected SMPs. Only a few tiny residual particles without surrounding inflammation were observed at week 8.

**Figure 6:**
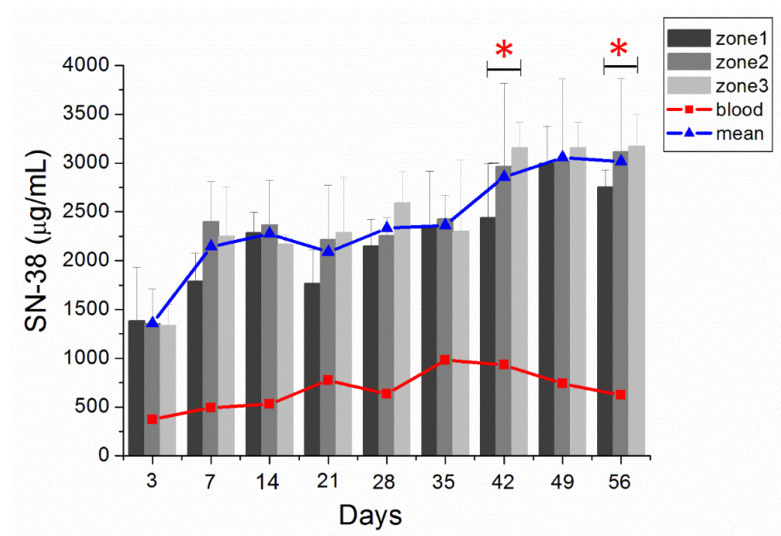
In vivo release. In vivo release of SN-38 from SMPs and concentrations in various zones of the brain parenchyma and blood (error bars indicate standard deviation). At day 3, the SN-38 concentration was higher in zone 1, whereas the SN-38 concentration was higher in zone 3 during the last three weeks. The difference between zone 1 and zone 3 was significant (*p* < 0.05) on days 42 and 56. (* indicated *p* < 0.05). The blue triangle indicated the mean SN-38 concentration of zones 1–3 and the red triangle denoted the mean concentration of blood.

The in vivo SN-38 level was also characterized for 8 weeks by using HPLC. Rats that died from massive blood loss or overdose of anesthetic during the perioperative period were excluded from the study. Accordingly, we acquired three rats on postinjection day (PID) 3, four on PID 7 (W1), four on PID 14 (W2), three on PID 21 (W3), three on PID 28 (W4), three on PID 35 (W5), three on PID 42 (W6), three on PID 49 (W7), and three on PID 56 (W8), for the analysis of in vivo drug concentrations. Figure 6 displays the SN-38 concentration curves obtained at the different zones of the brain and in plasma. The SN-38 concentration in the brain tissue had rapidly increased to a high level on PID 3 (1357.38 ± 139.21 µg/mL), reached the highest (3056.21 ± 72.56 µg/mL) on PID 49, and remained at high levels in the brain tissue for more than 8 weeks. The SN-38 concentration in plasma was much lower (ranging from 371.96 ± 285.77 to 981.78 ± 283.26 µg/mL).

Furthermore, the near-core area (zone 1) had a higher concentration than the core-distant area (zone 3) during the initial 3 days, but with no statistical significance. In the middle stage (weeks 3 and 4), the SN-38 concentrations at zones 1–3 were comparable. The SN-38 concentration was higher at zone 3 than at zone 1 in the later stage (weeks 6–8). The difference between zone 3 and zone 1 was significant at week 6 and week 8; *p*-values were 0.014 and 0.038, respectively.

### 3.6. Survival Rate

**Figure 7:**
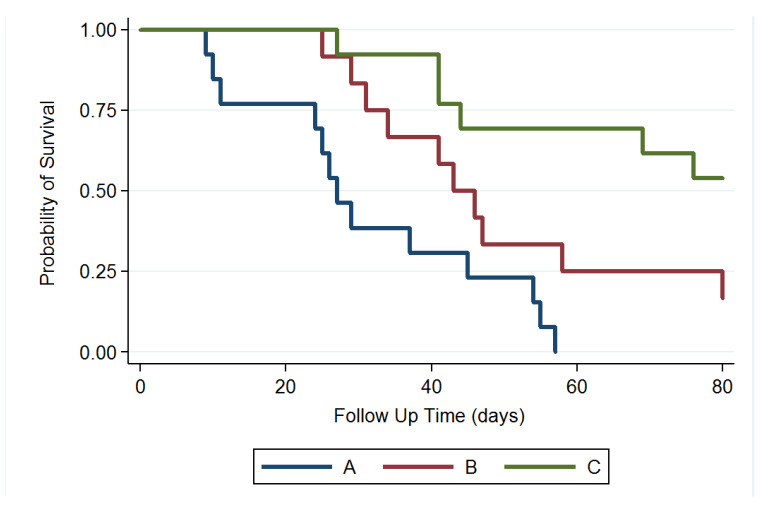
Survival curve. The median survival rate was significantly higher in group C than those in groups B and A; the survival rate was also higher in group B than that in group A; *p* = 0.028 for group B versus group A; *p* < 0.001 for group C versus groups B and A.

We excluded 4 rats that died within 48 h after the operation (perioperative period) and no rat died due to intracranial hemorrhage or abscess formation. MGs were successfully established and verified using T1- and T2-weighted MRI scanning in 43 rats. Thirty-eight MG-bearing rats (13, 12, and 13 in groups A, B, and C, respectively) were used for survival and tumor growth studies and 5 MG-bearing rats were used for pathologic studies (1, 2, and 2 in groups A, B, and C, respectively). In group A, more than half of the rats (7 of 13 rats) had died in week 4. At 8 weeks, 12 rats had died and only 1 survived. No rat in group A survived at the end of study (80 days). In group B, the rats received intratumoral injection of the Gliadel wafer mixed with 10 µL of DMSO, followed by oral TMZ. Five rats (of 12 rats) in group B had died in week 6, and 3 rats were alive at the end of study. In group C, 10 µL of SMPs/DMSO solution was injected intratumorally using a stereotaxic needle. Six rats had died at the end of the study; 53.85% (7/13) were alive, and no clear residual abscess was noted in the MRI images, in the three rat groups. Figure 7 displays the Kaplan–Meier survival curves for the representative survival times. SMPs significantly reduced the risk of mortality in the MG-bearing rats. The differences between groups A and C and between groups B and C were significant (*p* < 0.001), as was that between groups A and B (*p* = 0.028).

### 3.7. MRI and Tumor Volume

**Figure 8:**
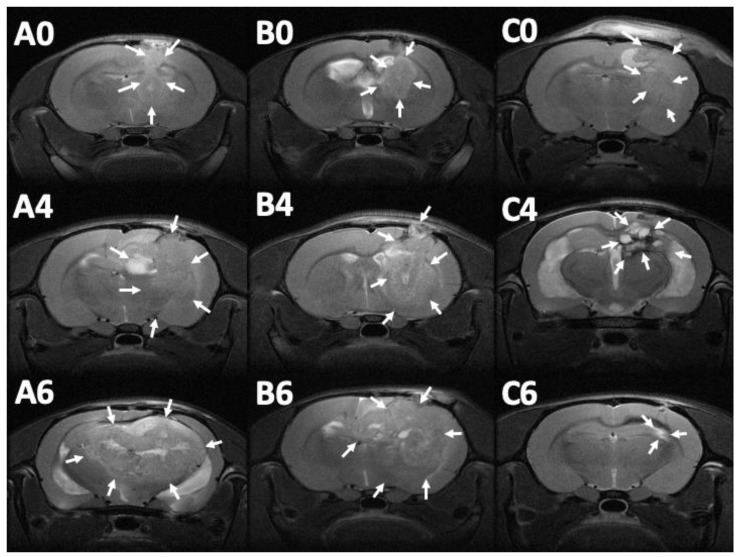
Serial magnetic resonance imaging. The label in the upper-left corner of each image denotes the number of posttreatment weeks. No significant primary discrepancy was noted among the brain tumors in groups A (A0), B (B0), and C (C0). The tumor in groups A and B increased promptly with an obvious mass effect. A rigorous midline shift with bilateral ventricle affection was found in group A (A4 and A6). The tumor expanded inwardly (B4) then traversed the midline (B6). The tumor size reduced gradually in group C, after injection of SMPs (C4 and C6), while no perifocal edema was noticed (the tumor areas are indicated by white arrows).

MRI images (T1- and T2-weighted) were obtained 12–14 days after injection of the F98 tumor cells into the cerebral parenchyma of rats, to confirm the successful creation of brain MG models. Serial brain MRI scans were obtained before treatment (0 day) and at 1, 2, 4, 6, and 8 weeks after treatment, in the three groups. Figure 8 shows the serial brain MRI scans of MG-bearing rats in the different therapeutic groups.

**Figure 9:**
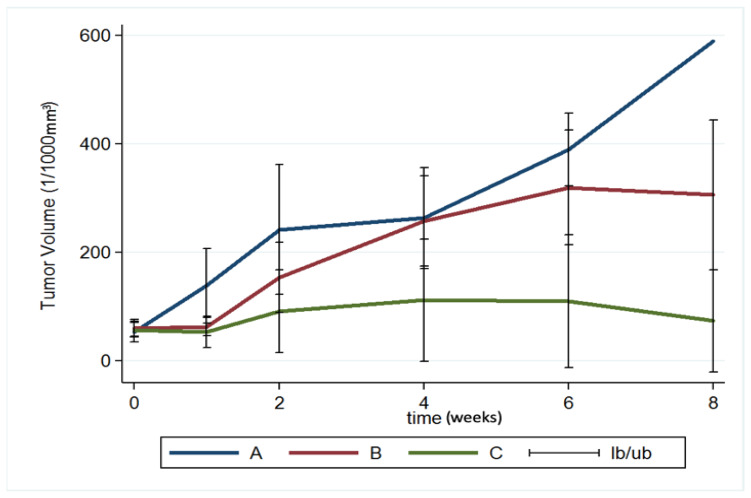
Brain tumor volume. A repeated-measures mixed model was used to evaluate brain tumor volume changes in the three groups. The brain tumor volume in groups A and B increased rapidly and was significantly greater than that in group C (group A vs. group C, *p* < 0.001; group B vs. group C, *p* < 0.001) (Ib/ub: inferior bound/ upper bound).

The brain tumor volumes increased rapidly in group A (injected with pure PLGA microparticles group). More than half of the rats (7/13) died in week 4, and the maximum tumor volume was 589.92 × 10^−3^ mm^3^ in the only surviving rat in week 8. In group B (treated with the Gliadel wafer), the mean tumor volume was 60.26 ± 15.75 and 62.43 ± 17.01 × 10^−3^ mm^3^ before treatment and at 1 week posttreatment, respectively. The tumor volume almost did not increase during the first week. Subsequently, the tumor enlarged rapidly and reached its maximum (319.08 ± 105.92 × 10^−3^ mm^3^) at week 6; 75% of the rats (9/12) died in week 8. Due to the death of rats with large brain tumors, the mean brain tumor volume of the remaining three rats in group B was only 305.84 ± 138.02 × 10^−3^ mm^3^. In group C (treatment with SMP injection), the mean tumor volume was 56.73 ± 13.00 × 10^−3^ mm^3^ before treatment, and slightly decreased to 52.89 ± 29.05 × 10^−3^ mm^3^ after 1 week, but the difference was nonsignificant. The tumor volume increased slightly and reached its maximum (111.47 ± 112.93 × 10^−3^ mm^3^) at week 4. The mean tumor volume decreased progressively after week 4, being 109.28 ± 122.66 and 73.14 ± 94.44 × 10^−3^ mm^3^ at weeks 6 and 8, respectively. The difference in tumor volume between week 0 and week 2 was nonsignificant (*p* = 0.74). Figure 9 shows the results of using a repeated-measures mixed model to evaluate the mean brain tumor volume of the three groups. Significant differences were discovered between groups A and C and between groups B and C (both *p* < 0.001). Nevertheless, no significant difference was found between groups A and B (*p* = 0.4).

### 3.8. Pathological Finding

**Figure 10:**
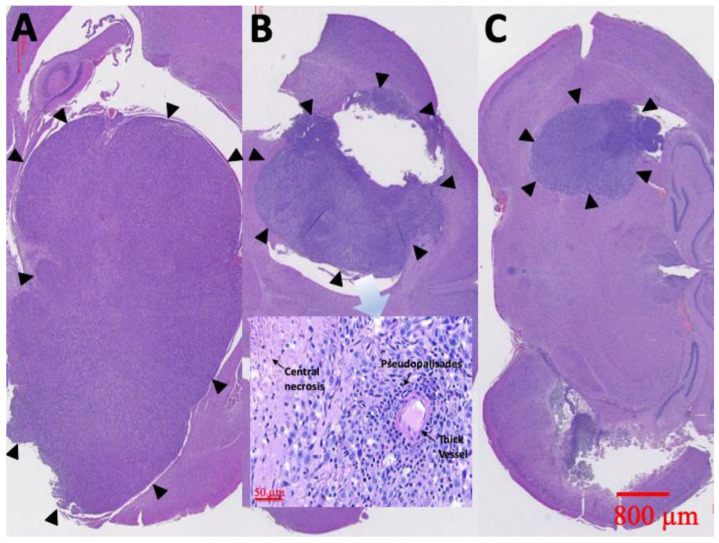
H&E staining. Diffuse karyorrhectic tumor cells with central necrosis were observed in groups A and B. The tumor area and central necrosis were much greater in group A than in group B. Small tumor area and less central necrosis were observed in group C (the tumor edges are indicated by black triangles).

At least one rat in each group was sacrificed and the brain tissues meticulously extracted through surgery, 6 weeks after treatment. Figure 10 displays the results of the H&E staining. Group A rats had a large tumor region containing diffused karyorrhectic tumor cells, with either microvascular proliferation with thick vascular walls or coagulation necrosis. In addition, considerable serpiginous necrosis with palisading near the necrotic foci was noted. Group B rats had respectably multinucleated tumor cells with a localized area but with endothelial proliferation and less pseudopalisading necrosis. By comparison, the tumor area in group C rats was distinctly smaller and confined.

**Figure 11:**
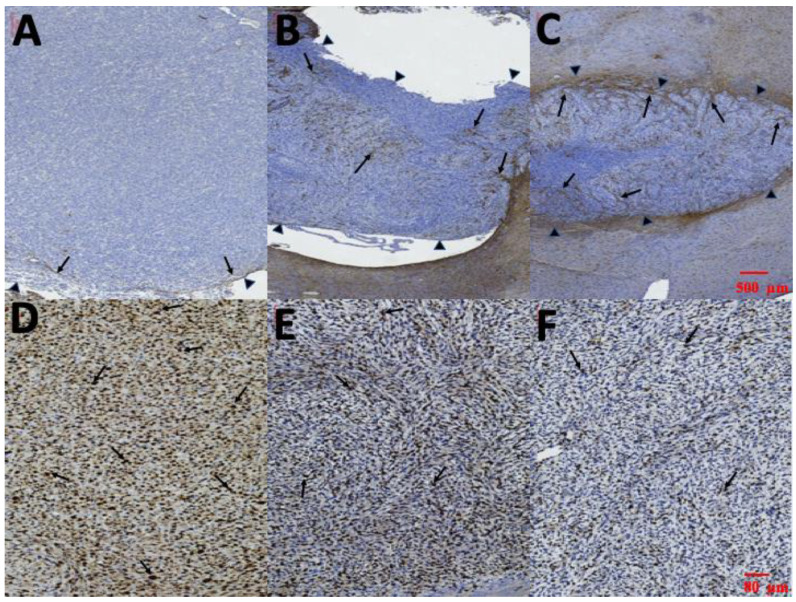
Glial fibrillary acidic protein (GFAP) and Ki-67. (**A**–**C**) Immunohistochemical staining of cytoplasmic processes for GFAP. Almost no intratumoral GFAP-positive cells were detected in group A; several thin GFAP-positive cells were found at the edge of the tumor in group B; and thick GFAP-positive cells with dendrites were noted intratumorally in group C (the tumor edges were indicated by small black triangles and the GFAP-positive cell were indicated by black arrows). (**D**–**F**) Ki-67 labeling index in each group; Ki-67 index in group A: 85.35% ± 9.21%; in group B: 63.35% ± 10.14%; and in group C: 29.56% ± 7.43%. (Ki-67 positive cells were indicated by black arrows.).

Immunocytochemical staining of cytoplasmic processes for GFAP is illustrated in Figure 11A–C. Limited GFAP-positive immunoreactivity was noted at the margin of tumors and no GFAP-positive immunoreactivity was detected within the tumor in group A (Figure 11A). However, several thin GFAP-positive glial cells were present in the margin and a few in the intratumor area in group B (Figure 11B). Thick intratumoral GFAP-positive glial cells with dendrites were determined in group C (Figure 11C). The Ki-67 labeling index in the three groups is illustrated in Figure 11D–E. The highest Ki-67 labeling index was in group A (85.35% ± 9.21%). MIB-1 immunoactivity demonstrated proliferation of 63.35% ± 10.14% in group B. The Ki-67 labeling index was lower in group C (29.56% ± 7.43%) than in groups A and B.

## 4. Discussion

This study successfully developed 7-ethyl-10-hydroxycamptothecia (SN-38)-embedded biodegradable poly[(d,l)-lactide-*co*-glycolide] microparticle (SMPs), stereotactically injected the particles into the brain parenchyma of healthy rats, and intratumorally injected into F98 glioma-bearing rats. The experimental results suggest that SN-38 was rapidly released after injection and its local (brain tissue) concentration remained much higher than that in the blood for more than 8 weeks. The injected SMPs also demonstrated significant therapeutic efficacy, with prolonged survival, retarded tumor growth, and attenuated malignancy.

Extensive infiltration of surrounding brain parenchyma prevents complete resection of tumor, whereas the BBB obstructs the adequate transport of most cancer cytotoxic agents, leading to the poor prognosis associated with MG [24]. New modalities that maximize the potential of cytotoxic drugs and reasonably prolong the overall survival of patients with MG are required. Advanced drug delivery systems are in use for the treatment of central nervous system (CNS) pathologies. Polymeric nanoparticles have been demonstrated to be one of the most promising vehicles for CNS drug delivery due to their potential for encapsulating bioactive agents, hence, protecting the agents from excretion and metabolism, and for delivering drugs across the BBB, without damaging the BBB [6,25,26]. In addition, nanoparticles provide protection to the incorporated bioactive agents against chemical or enzymatic degradation, increasing the likelihood that the active molecules reach the target lesion [6,25]. Polymeric particles that can be fabricated from synthetic or natural materials possess the beneficial properties of biodegradability, biocompatibility, and potential for functionalization, which are the prerequisite characteristics of drug carrier materials [6,25].

The results of animal studies [1,27] suggest that irinotecan exhibits antitumor activity against a broad panel of subcutaneous and intracranial human glioblastomas (GBMs), ependymomas, and medulloblastoma xenografts. Irinotecan shows potential efficacy and a significant activity against MG, when combined with other chemotherapeutic agents, including alkylating agents BCNU or TMZ [10,28]. A phase II study demonstrated that treatment of irinotecan combined with BCNU acted synergistically and was active and non-cross-resistant in patients with GBM, with recurrence after TMZ-based chemotherapy [1,29]. The combination of irinotecan and bevacizumab was strikingly active against recurrent MGs, with a significant but acceptable toxicity [30]. Irinotecan is a prodrug of SN-38 that exhibits some antitumor activity in patients with recurrent GBM, with a response rate of 0–17% in clinical trials [19,31,32]. The activity of irinotecan is dependent on the conversion ratio of irinotecan to SN-38 [19,32]. After systemic infusion, irinotecan is converted into its active metabolite, SN-38, which has a significantly higher cytotoxic potency [33]. An in vitro GBM cell study [34] suggested that SN-38 has a more potent antitumor effect on MG cells than irinotecan, regardless of multidrug resistance, and therefore can be considered a new chemotherapeutic agent for treatment of primary or recurrent MGs resistant to chemotherapy in humans. Direct use of SN-38 is theoretically effective for MG treatment; however, its clinical administration has been restricted mainly because SN-38 is water insoluble and highly toxic and thus cannot be delivered intravenously [19,32,33]. Additionally, SN-38 is unstable at body pH and has an extremely short half-life; an active form of SN-38 is converted into an inactive form within 9.5 min [35,36]. Furthermore, more than 90% of MGs eventually relapse at a location 1–2 cm from the original site, making local delivery an effective alternative [2,37]. Consequently, this study investigated the local delivery of SN-38 directly into the tumors and the surrounding cerebral tissue.

In an MG-bearing athymic/nude mice study, intratumoral administration of CPT-11 or SN-38 extended the average survival time of the MG-bearing animals from 22 days to 46 and 65 days, respectively [33]. Recent reports demonstrated that SN-38-loaded polymeric micelles or depots enhance and prolong the distribution of free SN-38 in vivo [35,38,39], and exert a potent antitumor effect against MG cells in vitro [38,40] and in animal models [19,35,41]. The effective release of SN-38 was reported to range from 14 h to 18 days [19,38,40,41]. In the present study, SN-38 was successfully loaded into PLGA microparticles [42] by using the electrospraying technique. The SMPs sustainably released a high concentration of SN-38 (approximately 10,000 µg/mL) to brain tissues, for more than 8 weeks, and the concentration in blood remained low. The longer treatment period used in this study theoretically enhanced the treatment efficacy and reduced resistance to chemotherapy. A major side effect of irinotecan or SN-38 is the gastrointestinal mucositis, an inflammation and ulceration of the gastrointestinal mucosa, mostly diagnosed on the basis of diarrhea occurrence [43,44]. No glioma-bearing rat treated with SMPs (group C) showed any noticeable sign of diarrhea. Additionally, PLGA is well-known to be biodegradable and biocompatible and has been among the most attractive polymeric candidates used to fabricate devices for drug delivery and tissue engineering applications. The erosion of PLGA is driven by hydrolysis, and the degradation products—glycolic and lactic acid—are nontoxic chemicals that can be eliminated by the human body via the Krebs cycle [7,45].

SMPs are injectable through stereotactic techniques; thus, tumors located in either nonsurgical or high-risk areas—such as the brain stem, midbrain, and deep brain—can be treated intratumorally with SN-38. The DMSO was employed as an assisting fluid for easy injection of microparticles into the brain tissue of rats via syringes. Other assisting fluids, such as biodegradable hydrogels might also be considered and selected. The experimental results obtained in this study suggested that the median survival time was significantly longer in group C than in group A (injected with PLGA/DMSO) or group B (treated with Gliadel wafer and oral TMZ). Moreover, the median survival time in group B was significantly longer than that in group A (*p* = 0.028). We constructed serial MRI images to estimate the variation in tumor volume. The tumor volumes in group A increased rapidly, whereas the tumor volumes did not change significantly in the first week in group B, and increased rapidly from week 2. The tumor volumes did not change significantly in the first 2 weeks in group C but subsequently grew slowly and reached their maximum (111.47 ± 112.93 × 10^−3^ mm^3^) at week 4, after which the tumor volumes decreased steadily. The differences in tumor volumes were significant (*p* < 0.001) between groups C and A and between groups C and B. However, the difference in tumor volume between groups A and B was nonsignificant. The findings demonstrated that the Gliadel wafer combined with oral TMZ had less therapeutic efficacy than the SMPs. Pharmacological studies have reported that Gliadel wafers can sustainably release BCNU and generate a high local BCNU concentration at a tumor site while minimizing systemic toxicity, with most of the drug released within the first 5–7 days [2,46].

GBM and astrocytoma originate from astroglial cells, and the number of cells expressing GFAP is inversely proportional to the extent of anaplasia. Loss of GFAP expression is frequently observed in high-grade astrocytoma [47,48]. Ki-67 is a nuclear antigen, the expression of which is the simplest and the most reliable means of assessing astrocytic tumor proliferative potential, and provides crucial prognostic information correlated with the grade of human astrocytomas [48,49]. In the present study, H&E staining indicated a large tumor area with diffuse multinucleated tumor cells and considerable necrotic foci in group A. The tumor area and necrotic foci were less in the SMP-treatment group (group C). Serial brain MRI also demonstrated that the tumor volumes decreased in the SMP-treatment group, whereas only temporary retardation of tumor growth was noticed in the first week in group B, and rapid tumor growth was discovered in group A. GFAP expression within the tumor was negative in group A. Several sparse GFAP-positive cells were observed within the tumor in group B, and numerous thick GFAP-positive cells with dendrites within the tumor area were discovered in group C. The Ki-67 index was the highest in group A and lowest in group C. The GFAP expression findings were consistent with those for the Ki-67 index. In addition, the pathological results demonstrated that the malignancy of the created MGs was clearly attenuated after treatment with SMPs. The median survival rate was significantly higher in group C than in the other groups, with that in group B being the next highest and that in group A being the lowest. The differences between groups C and B, groups C and A, and groups B and A were all significant (*p* < 0.001, <0.001, and 0.028, respectively).

## 5. Conclusions

The present study provided experimental evidence that SMPs could be efficiently administered into the brain through stereotactic techniques. The biodegradable SMPs sustainably delivered high concentrations of SN-38 for more than 8 weeks with temporary inflammation in the brain tissue. The F98 MG-bearing rats treated with SMPs had advantages in terms of the restricted and retarded tumor growth, extended survival, and attenuated malignancy. The SMPs exhibited favorable activity against MG in the F98 MG-bearing rats, when used at concentrations lower than those causing dose-limiting systemic or neurological toxicity to the normal brain. The results implied that SMPs have a potential efficacy for interstitial chemotherapy and are a favorable alternative to the current therapeutic options for MG.
